# An Enhancer Element Harboring Variants Associated with Systemic Lupus Erythematosus Engages the *TNFAIP3* Promoter to Influence A20 Expression

**DOI:** 10.1371/journal.pgen.1003750

**Published:** 2013-09-05

**Authors:** Shaofeng Wang, Feng Wen, Graham B. Wiley, Michael T. Kinter, Patrick M. Gaffney

**Affiliations:** 1 Arthritis and Clinical Immunology Research Program, Oklahoma Medical Research Foundation, Oklahoma City, Oklahoma, United States of America; 2 Free Radical Biology and Aging Research Program, Oklahoma Medical Research Foundation, Oklahoma City, Oklahoma, United States of America; University of Texas Southwestern Medical Center, United States of America

## Abstract

Functional characterization of causal variants present on risk haplotypes identified through genome-wide association studies (GWAS) is a primary objective of human genetics. In this report, we evaluate the function of a pair of tandem polymorphic dinucleotides, 42 kb downstream of the promoter of *TNFAIP3*, (rs148314165, rs200820567, collectively referred to as TT>A) recently nominated as causal variants responsible for genetic association of systemic lupus erythematosus (SLE) with tumor necrosis factor alpha inducible protein 3 (*TNFAIP3*). *TNFAIP3* encodes the ubiquitin-editing enzyme, A20, a key negative regulator of NF-κB signaling. A20 expression is reduced in subjects carrying the TT>A risk alleles; however, the underlying functional mechanism by which this occurs is unclear. We used a combination of electrophoretic mobility shift assays (EMSA), mass spectrometry (MS), reporter assays, chromatin immunoprecipitation-PCR (ChIP-PCR) and chromosome conformation capture (3C) EBV transformed lymphoblastoid cell lines (LCL) from individuals carrying risk and non-risk *TNFAIP3* haplotypes to characterize the effect of TT>A on A20 expression. Our results demonstrate that the TT>A variants reside in an enhancer element that binds NF-κB and SATB1 enabling physical interaction of the enhancer with the *TNFAIP3* promoter through long-range DNA looping. Impaired binding of NF-κB to the TT>A risk alleles or knockdown of SATB1 expression by shRNA, inhibits the looping interaction resulting in reduced A20 expression. Together, these data reveal a novel mechanism of *TNFAIP3* transcriptional regulation and establish the functional basis by which the TT>A risk variants attenuate A20 expression through inefficient delivery of NF-κB to the *TNFAIP3* promoter. These results provide critical functional evidence supporting a direct causal role for TT>A in the genetic predisposition to SLE.

## Author Summary

A key objective of human genetics is the identification and characterization of variants responsible for association with complex diseases. A pair of single nucleotide polymorphisms (rs148314165, rs200820567) 42 kb downstream from the promoter of *TNFAIP3*, have been proposed as the variants responsible for association with systemic lupus erythematosus based on comprehensive genetic and bioinformatic analyses. *TNFAIP3* encodes for the ubiquitin-editing enzyme, A20, which plays a central role in maintaining immune system homeostasis through restriction of NF-κB signaling. Cells that carry this risk haplotype express low levels of *TNFAIP3* compared to cells carrying the nonrisk haplotype. How the risk alleles of rs148314165 and rs200820567 might influence low *TNFAIP3* expression is unknown. In this paper, we demonstrate that these variants reside in an enhancer element that binds NF-κB and SATB1 enabling the interaction of the enhancer with the *TNFAIP3* promoter through long-range DNA looping. Impaired binding of NF-κB directly to the risk alleles or shRNA-mediated knockdown of SATB1 inhibits interaction of the enhancer with the *TNFAIP3* promoter resulting in reduced A20 expression. These results clarify the functional mechanism by which rs148314165 and rs200820567 attenuate A20 expression and support a causal role for these variants in the predisposition to autoimmune disease.

## Introduction

*TNFAIP3* encodes A20, an ubiquitin-editing enzyme with a key role in negatively regulating NF-κB pathway activity downstream of activating cell surface receptors [1]–[4]. Murine models have been illustrative in demonstrating the importance of A20 in limiting immune responses. For example, mice globally deficient for A20 experience widespread organ inflammation and perinatal death [2]. Mice with A20 deficiency localized to B lymphocytes demonstrate enhanced responses to toll-like receptor, B cell receptor and CD40 receptor stimulation, elevated numbers of plasma and germinal center B cells and immune complex deposition in the kidneys [5]–[7]. Mice with A20 deficient dendritic cells excrete high levels of proinflammatory cytokines and spontaneously activate lymphoid and myeloid cells resulting in lymphadenopathy and splenomegaly [8]. In humans, at least 8 GWAS in 5 autoimmune diseases have reported genome wide significant associations with variants in the vicinity of *TNFAIP3* and others have reported suggestive association [9]–[18]. Lymphoid malignancies such as diffuse large B-cell lymphoma, marginal zone lymphoma, follicular lymphoma, MALT lymphoma and Hodgkin lymphoma, often carry deletions or inactivating point mutations in *TNFAIP3* suggesting a role for *TNFAIP3* as a tumor suppressor [19]–[23]. These observations, in both animal models and human subjects, highlight the need to clarify how SLE associated genetic variants in the *TNFAIP3* locus may influence the maintenance of immune homeostasis toward the development of autoimmunity.

SLE is a severe autoimmune disease characterized by immune complex mediated inflammation of target organs (kidney, brain, skin), high titer autoantibody production and dysregulated interferon pathway activity. There is no curative therapy for SLE. Patients are most often treated with broad-spectrum immunosuppressive agents, the side effects of which contribute to the already considerable morbidity of the disease. Ongoing efforts to better understand the genetic, immunologic and environmental factors that contribute to SLE holds promise for future advances in the prognosis, diagnosis and therapy. To that end, genetic studies have convincingly identified over 30 loci associated with SLE [24], [25]. However, for most loci, the variants responsible for association (causal variants) still await identification. Of the three known independent genetic effects reported in the *TNFAIP3* locus, the most consistently replicated is a ∼100 kb risk haplotype that spans the *TNFAIP3* gene body [9],[15],[17],[26]. This risk haplotype has been observed in SLE subjects of both European and Asian ancestry but has not been convincingly detected in SLE subjects of African origin [27]. Genetic studies in other autoimmune diseases including systemic sclerosis, Sjogren's syndrome and rheumatoid arthritis indicate that they likely share this risk haplotype with SLE [28]–[31].

A coding variant, rs2230926, which results in a phenylalanine to cysteine substitution at position 127 in exon 3 of *TNFAIP3*, has been used as a marker of the *TNFAIP3* risk haplotype in genetic studies. Even though the risk allele (G) of rs2230926 is associated with decreased potency for inhibiting NF-κB signaling compared to the nonrisk (T) allele using in-vitro transfection assays [26], the evidence for this polymorphism as a causal variant is not convincing. The primary evidence supporting this conclusion comes from the observation that the G allele of rs2230926 has a minor allele frequency of 30–40% in African American SLE and yet no significant association with SLE is observed in this population [27]. Therefore, while this variant may alter A20 function, is not likely a causal variant.

We recently proposed a pair of tandem polymorphic dinucleotides (rs148314165, rs200820567) located in the genomic DNA 30 kb telomeric of *TNFAIP3* to be the most likely candidate variants responsible for association with SLE based on transpopulation differences in LD between in associated (European and Asian) and non-associated (African American) populations and bioinformatic annotation demonstrating that these variants are located in an evolutionarily conserved region of regulatory significance [27]. The TT>A risk alleles are carried on a risk haplotype that is associated with hypomorphic expression of *TNFAIP3* transcripts and A20 protein [27]. The mechanism by which the TT>A risk variants might influence the hypomorphic expression of A20 is unknown and serves as key evidence for assigning causality. In this study, we demonstrate that the TT>A variants are located in a functional enhancer element that binds NF-κB and SATB1 and the risk alleles of TT>A directly lead to reduced expression of A20 by their inability to effectively bind and deliver NF-κB to the *TNFAIP3* promoter through long-range DNA looping.

## Results

### The TT>A risk alleles bind NF-κB subunits with reduced affinity

**Figure 1:**
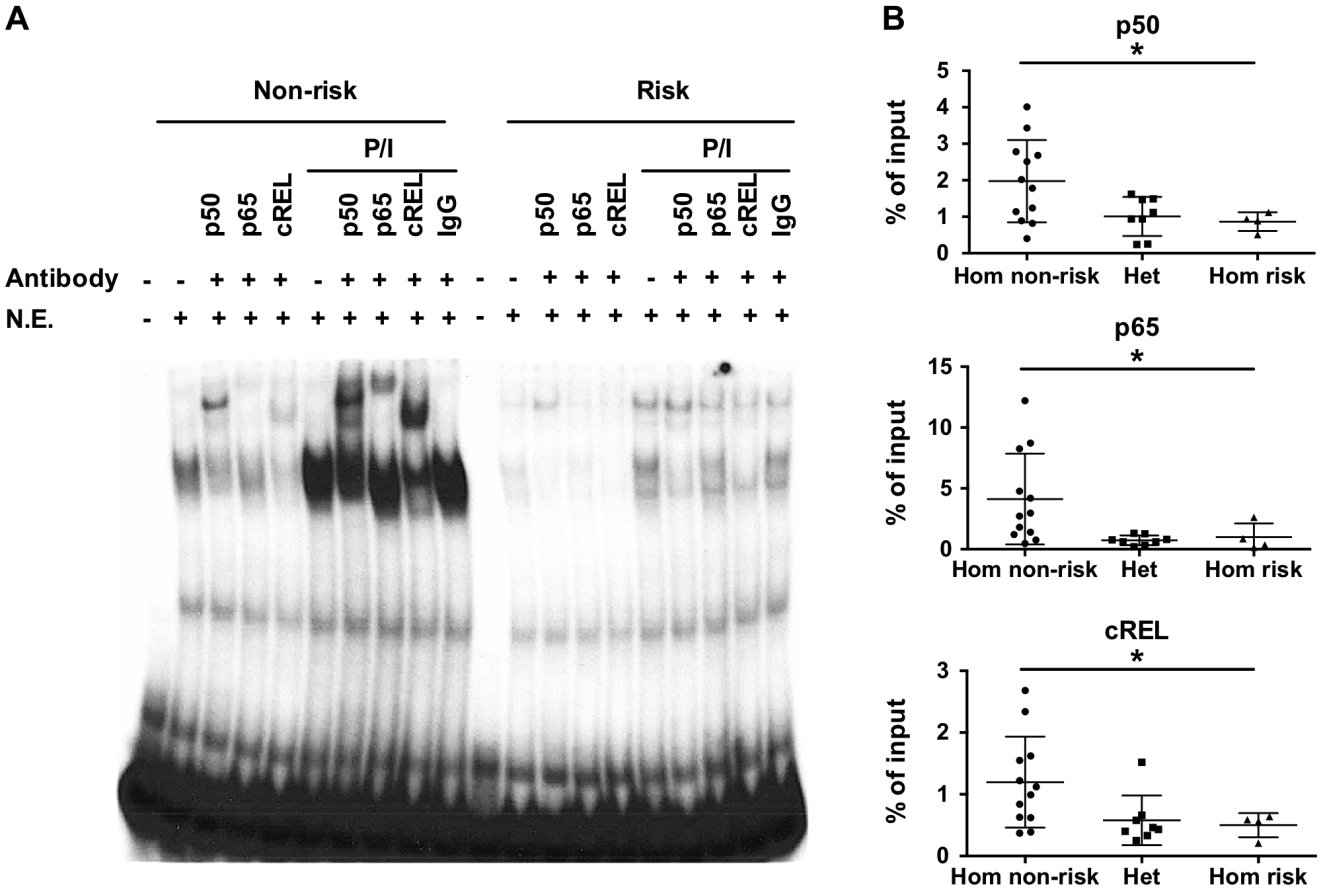
The TT>A variants result in reduced binding of a nuclear protein complex that contains NF-κB subunits. a. EMSA was performed with radiolabeled nucleotides containing the non-risk (TT) (40 bp) and risk (-A) (39 bp) polymorphisms. Nuclear extracts were derived from EBV transformed B cell lines at rest or stimulated with P/I and in the presence or absence of the indicated NF-κB subunit antibodies. Rabbit IgG was used as the isotype control. N.E. -nuclear extracts. b. ChIP-pPCR was performed using EBV transformed B cell lines (-A/-A = 4; TT/-A = 8; TT/TT = 12) stimulated with P/I. ChIP was performed with antibodies specific against NF-κB p50, p65, and cRel subunits, followed by qPCR with primers neighboring TT>A polymorphic region. Statistical comparisons were made using one-way ANOVA, * indicates p<0.05.

The variants rs148314165 (-T) and rs200820567 (T>A), referred to as TT>A, are located in a conserved region of genomic DNA that exhibits open chromatin, epigenetic marks of active enhancers and interaction with several transcription factors including NF-κB (Figure S1). Since the *TNFAIP3* gene product, A20, functions to restrict NF-κB signaling, we focused on characterizing the binding of NF-κB subunits to the region. We used the UniProbe database [32] to evaluate the region defined by the ENCODE NF-κB binding signal (chr6:138,229,889–138,230,230, hg19) for the presence of NF-κB binding motifs. Three NF-κB sites were identified, with the first site incorporating the TT>A variant (Figure S2A). Our previous work [27] used an EMSA probe that included both the TT>A site and the second NF-κB site, so we redesigned the probes to include only the TT>A site in order to isolate the contribution to the EMSA signal to this site. EMSA demonstrated stimulus enhanced binding of a nuclear protein complex to the 40 bp non-risk (TT) probe using nuclear extracts from EBV transformed B cells (Figure 1A). Complex formation was reduced when the risk allele (-A) was introduced into the probe sequence, suggesting that the risk allele alters the binding affinity of this complex (Figure 1A). Super shift experiments demonstrated NF-κB subunits NFKB1 (p50), cREL, RELA (p65) in the nuclear protein complex (Figure 1A). Similar results were also observed using nuclear extracts from the monocytoid cell line, THP1 (Figure S3). The specificity of our EMSA probes was confirmed by competition with unlabeled probe (Figure S4).

To validate the EMSA results using an orthogonal approach, we performed chromatin-immunoprecipitation (ChIP) followed by quantitative PCR using EBV cell lines carrying all three genotype combinations (TT/TT, TT/-A, -A/-A) at the TT>A polymorphic site. We observed significantly lower enrichment as a percentage of input DNA from cell lines carrying the risk allele (TT/-A or -A/-A) for all three NF-κB proteins (p<0.05) (Figure 1B). Control experiments using antibodies to acetyl-histone H3 (positive control) or rabbit isotype control IgG (negative control) demonstrated no specific differences in enrichment as expected (Figure S5). Together with the EMSA data, these data confirm that NF-κB subunits bind to this regulatory element following cell stimulation and that this binding is impaired by the presence of the risk (-A) variant.

### The TT>A variants are located in an enhancer element

**Figure 2:**
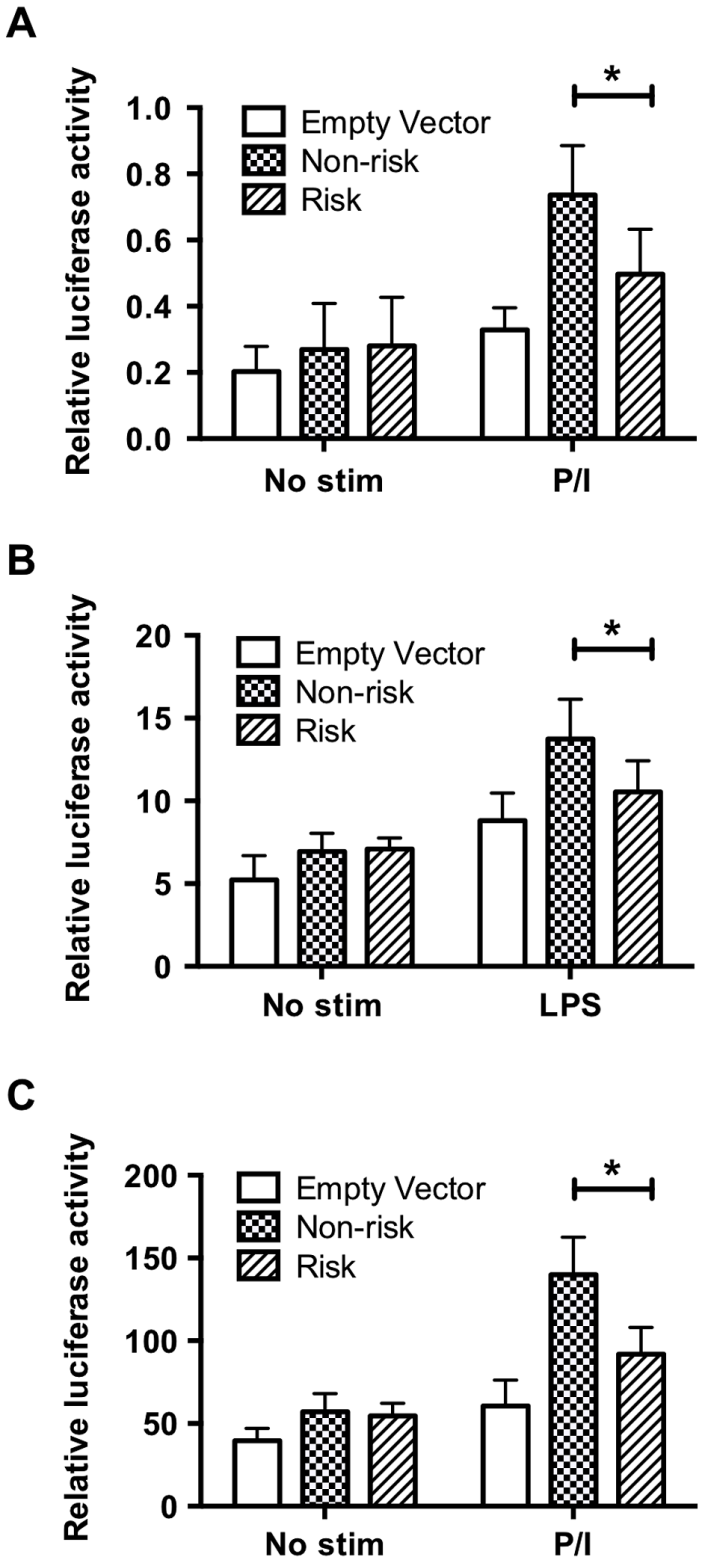
The regulatory element containing the TT>A variants demonstrates enhancer activity. Sequences carrying risk (-A) and non-risk (TT) polymorphisms were cloned upstream of a minimal thymidine kinase promoter luciferase construct to measure luciferase activation following transient transfection and stimulation. a. HEK293T cells stimulated with P/I for 48 hours. b. THP1 cells stimulated with LPS for 48 hrs. c. THP1 cells stimulated with P/I for 48 hours. Statistical comparisons were performed using a Student's *t*-test of three biological independent experiments, * indicates p<0.05.

Given the substantial distance of the TT>A polymorphism from the *TNFAIP3* promoter, the stimulus dependent recruitment of NF-κB subunits to the site and ENCODE histone marks, we hypothesized that this element may function as an enhancer. To test this hypothesis, we cloned the non-risk (TT) or risk (-A) variants and approximately 168 bases of flanking sequence (chr6:138,229,810–138,230,149; hg19) that included the two NF-κB sites downstream of TT>A (Figure S2A) into a minimal TK promoter construct. Plasmids were transfected into HEK293T or THP1 cells followed by stimulation with PMA/ionomycin (PI) (HEK293T and THP1) or LPS (THP1 only). Compared to the minimal TK promoter alone, we observed a significant increase in luciferase activity following stimulation with PI or LPS for both non-risk (TT) and risk (-A) plasmids suggesting that this regulatory element functions in a manner consistent with an enhancer (Figure 2). However, the risk (-A) construct produced significantly lower levels of luciferase activity compared with the non-risk (TT) construct (Figure 2). Similar differences were also observed using constructs lacking the two downstream NF-κB sites (Figure S6). These results demonstrate that the regulatory element containing the TT>A variant functions as an enhancer and the presence of the risk (-A) allele, which binds NF-κB with reduced affinity, impairs enhancer function.

### DNA sequence adjacent to TT>A binds SATB1

To identify other proteins that interact with the TT>A enhancer we affinity purified proteins bound to biotinylated probes used in our EMSA experiments followed by gel purification and mass spectroscopy (MS) analyses. We identified a band that migrated between 80 and 100 kD pulled down by both the risk (-A) and non-risk (TT) probes that was not observed using a control probe with a scrambled sequence (Figure S7A). MS results from three separate experiments identified this protein as special AT-rich binding protein 1 (SATB1) (Figure S7B). Inspection of the probe sequences revealed a SATB1 binding motif (AATAA) adjacent to the NF-κB (Figure S2B). We confirmed the presence of SATB1 by western blotting using eluted protein from affinity purification (Figure S7C) and EMSA supershift (Figure S3). No differences in affinity for the risk versus non-risk probes were observed suggesting that the TT>A polymorphism may not directly influence the binding affinity of SATB1.

### The TT>A enhancer interacts with the *TNFAIP3* promoter by DNA looping

**Figure 3:**
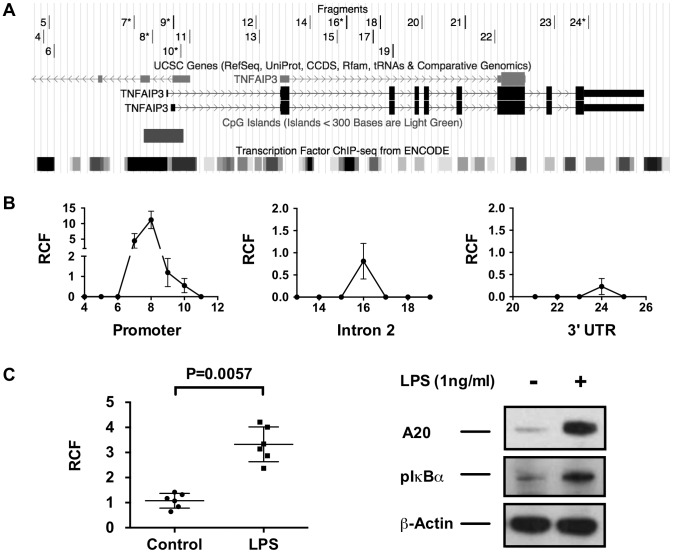
3C analysis demonstrates long-distance interactions between the TT>A enhancer and the *TNFAIP3* gene region. a. The track on the top is the location of the *TNFAIP3* primers used to identify potential amplified interaction fragments tested by 3C. Primers 6–10, 16 and 24 produced signals and are marked with asterisks. The middle and bottom track shows the genomic region of *TNFAIP3* with the location of the promoter CpG island and ENCODE defined transcription factor binding sites. b. Increased relative interaction frequencies (RCF) were detected in three regions of *TNFAIP3*, the promoter, intron 2 and the 3′ untranslated region. c. Stimulation of THP1 cells with LPS results in increased 3C interactions between the TT>A enhancer and the *TNFAIP3* promoter along with a concomitant increase in A20 expression and IκBα phosphorylation. Shown is a representative blot from 3 independent experiments. Statistical differences were determined using Student's *t*-test.

A primary function of SATB1 is to facilitate long-range gene transcription through chromatin remodeling and DNA looping [33], [34]. To determine if long-range DNA looping occurs between the TT>A enhancer and the *TNFAIP3* promoter, we performed chromatin conformation capture (3C) using a series of PCR primers (Figure 3A) distributed across key regulatory elements in the genomic sequence upstream of *TNFAIP3* and in the *TNFAIP3* promoter and gene body (Figure 3A). We detected interaction from three regions of *TNFAIP3* (Figure 3B). The largest and most reproducible relative crosslinking frequency (RCF) was located in the *TNFAIP3* promoter in a region enriched for transcription factor binding sites. Importantly, the peak RCF detected by primer 8, is near a region of the promoter previously reported to bind NF-κB and stimulate transcription of *TNFAIP3* [35]. The second highest RCF (primer 16) was located in the second intron, again in a region enriched in transcription factor binding sites but was approximately 10 fold weaker than the promoter signal. The third and weakest RCF (primer 24) was located in the 3′ untranslated region and was half the magnitude of the second signal. To verify these results we tested other cell lines derived from a variety of lineages. The RCF in the promoter of *TNFAIP3* was reproducibly observed in all cell types evaluated (Figure S8). Stimulating THP1 cells with LPS for 2 hours produced a significant increase in the RCF detected between the TT>A enhancer and the *TNFAIP3* promoter and was accompanied by a concomitant increase in A20 protein and phospho-IκBα expression (Figure 3C). These results reveal a novel mechanism of transcriptional regulation whereby the TT>A polymorphic enhancer delivers an NF-κB payload to the *TNFAIP3* promoter leading to increased expression of A20.

### SATB1 knockdown inhibits TT>A enhancer/*TNFAIP3* promoter interactions

**Figure 4:**
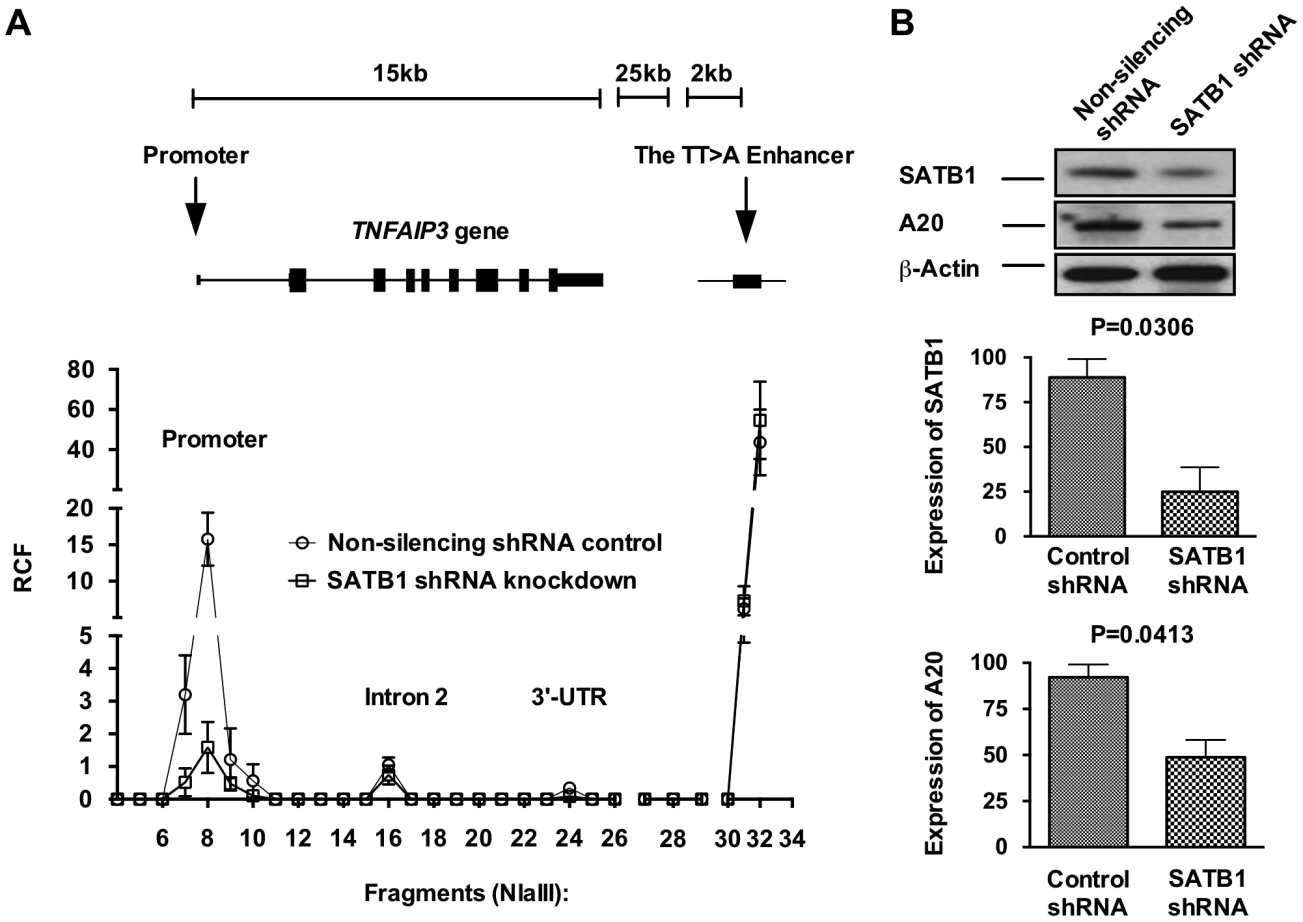
SATB1 is required for the TT>A enhancer-promoter interaction and *TNFAIP3* transcription. a. Shown is a representative 3C assay from 3 independent experiments in HEK293T cells. Relative crosslinking frequencies (RCF) were normalized to both *GAPDH* loading control and a *TNFAIP3* BAC clone and plotted according to its location to *TNFAIP3* gene and the TT>A enhancer. High local interaction frequencies near the TT>A enhancer serve as a positive control (fragments 30–34). b. The top panel shows the protein expression levels of SATB1 and A20 were detected using Western blots with antibodies against SATB1 and A20, respectively. β-actin was used as loading control. The bottom two panels show densitometer quantification of the relative expression of SATB1 (middle panel) and A20 (lower panel) normalized to β-actin. Error bars represent standard error of the mean from 3 independent experiments. Differential protein levels between SATB1 knockdown and non-silencing shRNA control were calculated using Student's *t*-test.

We next tested whether the RCF between the TT>A enhancer and *TNFAIP3* promoter was dependent on expression of SATB1 (Figure 4). SATB1 expression was inhibited using a SATB1 specific shRNA construct transfected into HEK293T cells followed by 3C (Figure 4B). Results from these experiments demonstrated a significant reduction in the RCF between the TT>A region and the *TNFAIP3* promoter (Figure 4A) with inhibition of SATB1 expression, accompanied by a reduction in A20 protein expression (Figure 4B). These data suggest that the TT>A polymorphism modulates *TNFAIP3* transcription through a SATB1 mediated long range looping mechanism and that interfering with looping leads reduced *TNFAIP3* transcription and A20 protein expression.

### TT>A risk alleles demonstrate fewer interactions with the *TNFAIP3* promoter

**Figure 5:**
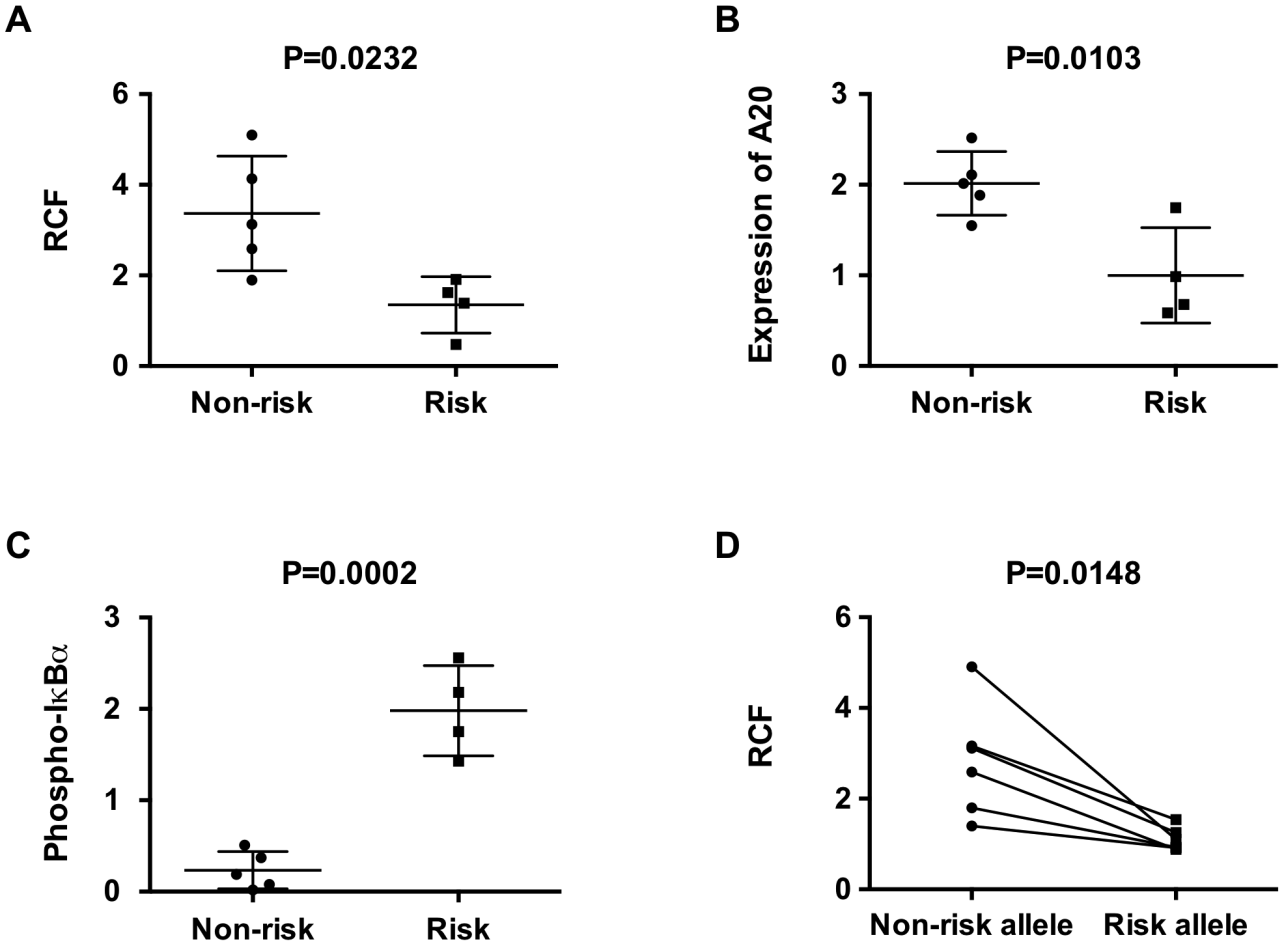
The TT>A risk variant exhibits reduced enhancer-promoter interaction frequencies and lower expression of *TNFAIP3*. a. 3C-qPCR assays were performed in EBV-transformed B cells homozygous for either risk or non-risk alleles in the TT>A enhancer; crosslinking frequencies between the enhancer and the promoter were normalized to *GAPDH* control. b, c. Protein expression of A20 and phospho-IκBα in homozygous cell lines were determined using Western blot and were normalized to loading control β-actin. Plots represent the relative expression of densitometric measurements for each cell line. d. Allele-specific 3C sequencing results of EBV cell lines heterozygous for the TT>A alleles. The read counts for each paired allele from 3C samples were normalized to the read counts for each allele from parallel analysis of input genomic DNA not subjected to 3C. Comparisons were made between non-risk and risk alleles for each individual. Statistical differences were calculated using paired *t*-test.

Having established that the TT>A enhancer interacts with the *TNFAIP3* promoter and that inhibition of looping results in reduced A20 expression, we wanted to determine if the autoimmunity associated risk allele (-A) influenced the interaction frequency. Evaluation of crosslinking frequencies in resting EBV transformed B cell lines demonstrated a significantly higher RCF in homozygous (TT/TT) non-risk cells compared to homozygous risk cells (-A/-A) (Figure 5A). This was accompanied by reduced expression of A20 (Figure 5B) and increased basal NF-κB pathway activity as measured by IκBα phosphorylation in homozygous risk cell lines (Figure 5C). To validate these results and to reduce potential bias in the detection of the RCF due to the multi-step 3C protocol, we developed a sequencing-based read-counting allele specific 3C assay that tallies the number of ligation products occurring from each allele in heterozygote (TT/-A) cell lines. Using this method, we again detected significantly fewer looping interactions produced from the risk allele (-A) compared with the non-risk (TT) allele thus confirming our results in homozygous cell lines (Figure 5D). These results suggest that reduced binding of NF-κB to the TT>A risk allele results in less interaction with the *TNFAIP3* promoter and lower expression of A20 protein.

## Discussion

In this report, we describe the functional characterization of the TT>A variants that are associated with human SLE in the region of *TNFAIP3* on chromosome 6q23 for which previous genetic and bioinformatics analyses suggest they are likely to be causal variants. Identification and functional characterization of causal variants responsible for disease predisposition is a fundamental goal of human genetics. Even though GWAS have identified thousands of variants reproducibly associated with hundreds of complex genetic diseases [36] only a small fraction of these variants are presumed to be causal. This is due to the presence of linkage disequilibrium (LD) in the human genome, which streamlines GWAS discovery but renders causal variants statistically indistinguishable from noncausal variants on the same haplotype. Isolating causal from noncausal variants is a formidable task and most often involves a combination of genetic (finemapping, resequencing, imputation) and bioinformatic (variant annotation, modeling building) approaches. Typically, the end result of these studies is a prioritized list of variants that must be systematically evaluated for allelic differences in biological function. Our results provide a functional explanation for the genetic association between SLE and the minor alleles rs148314165 and rs200820567 and compelling evidence that these variants are causal variants for this risk haplotype.

The *TNFAIP3* locus exhibits complex genetic architecture with multiple variants demonstrating significant genetic associations across multiple autoimmune phenotypes. Our study focuses specifically on the ∼100 kb risk haplotype that spans the *TNFAIP3* gene body first identified in SLE. These variants likely also explain the association signals detected in other autoimmune diseases testing variants in strong LD with rs148314165 and rs200820567 in subjects of European or Asian background including the coding variant rs2230296. The TT>A risk haplotype is, however, distinct from a *TNFAIP3* risk effect first reported in psoriasis and marked by SNP rs610604 [37] located in the sixth intron of *TNFAIP3*. The correlation in European and Asian populations between rs610604 and rs7749323, a perfect proxy for TT>A, is low (r^2^<0.1), indicating that psoriasis is likely associated with different causal variants. Our results also do not explain both risk and protective associations located ∼200 kb upstream of *TNFAIP3* reported most robustly in rheumatoid arthritis [18], [38] and celiac disease [39] but also in SLE and inflammatory bowel disease. It is possible that variants associated with these diseases will impact other uncharacterized enhancers in a manner similar to that described here for the TT>A enhancer. Alternatively, a long non-coding RNA encoded on the negative strand adjacent to *TNFAIP3 (AK124173)* shares the same promoter region and may influence *TNFAIP3* expression or translation through as yet to be defined mechanisms. Despite the uncertainties, further genetic and functional characterization in appropriate disease subjects will be required to clarify the causal variants responsible for these associations and the mechanisms of *TNFAIP3* function that they govern.

In summary, these results reveal a novel mechanism of *TNFAIP3* transcriptional regulation whereby the TT>A enhancer element delivers NF-κB to the *TNFAIP3* promoter through long-range DNA looping thus stimulating A20 protein expression. The SLE associate TT>A risk alleles, through their inability to effectively deliver NF-κB to the *TNFAIP3* promoter, impair A20 expression leading to enhanced NF-κB pathway activity and predisposition to autoimmune disease. Clarifying the functional basis by which DNA sequence variants such as these perturb cellular pathways toward the disease state will be crucial in translating GWAS discoveries into knowledge that can improve human health.

## Materials and Methods

### Ethics statement

Written informed consent was obtained from all study participants. The overall study was approved by the IRB of the Oklahoma Medical Research Foundation (OMRF).

### Antibodies and cell lines

THP-1, U937, Jurkat and Daudi were purchased from ATCC. EBV-transformed B cell lines were obtained from the Lupus Family Registry and Repository (OMRF) with IRB approval. EBV cell lines were selected using genotype data corresponding to the TT>A variant proxy marker rs7749323. Cell lines were maintained in RPMI 1640 medium supplemented with 10% FBS, penicillin, streptomycin, L-glutamine and 55 µM β-mercaptoethanol. Lipopolysaccharide (LPS), Phorbol myristate acetate and Ionomycin (P/I) were purchased from Sigma-Aldrich. The following antibodies were used in this study: Anti-phospho-IκBα, anti-SATB1, anti-β-actin and anti-GAPDH (Cell signaling Inc., Danvers, MA), Anti-A20 antibody (Ebioscience Inc., San Diego, CA), anti-p50, anti-p65, and anti-cRel antibodies (GeneTex Inc., Atlanta, GA).

### Electrophoretic mobility shift assays

40 base pair (non-risk) or 39 bp (risk) DNA probes were synthesized and end-labeled with (γ-^32^P) adenosine triphosphate (MP Biomedicals Int.) using T4 polynucleotide kinase (Invitrogen, Grand Island, NY). Nuclear protein extracts were prepared from cells stimulated with LPS (1 ug/mL) or P/I (50 ng/ml, 500 ng/ml) for 2 hours and incubated for 25 min at 37°C with labeled probes in binding buffer (1 ug poly dI-dC, 20 mM HEPES, 10% Glycerol, 100 mM KCl, and 0.2 mM EDTA, pH 7.9). DNA-protein complexes were resolved on non-denaturing acrylamide gels. Supershift assays were performed by adding 80–100 ug of anti-p50, p65, c-Rel antibodies or Rabbit IgG isotype control antibody (Alpha Diagnostic Int. Inc.) to the mixture followed by incubation at room temperature for 15 min prior to adding labeled probe.

### Chromatin immunoprecipitation (ChIP) and Quantitative-PCR (qPCR)

ChIP assays were performed using the Magna ChIP A kit (Millipore, Billerica, CA) according to the manufacturer's recommendations. In brief, 1×10^7^ EBV transformed B cells were treated with P/I (50 ug/ml, 500 ng/ml) in 10 ml growth medium for 2 hours and were cross-linked with 1% formaldehyde. Nuclei were isolated and sonicated in 500 ul of lysis buffer with a Covaris S1 sonicator (Woburn, MA). Fifty microliters of chromatin-protein complexes were immunoprecipitated overnight at 4°C by mild agitation with antibodies specific for p50, p65, cRel, acetyl-histone H3 (positive control) (Millipore, Billerica, CA), or normal rabbit IgG (negative control) (Millipore, Billerica, CA). DNA was eluted from the immunoprecipitated chromatin complexes, reverse-crosslinked, purified by Agencourt AMPure XP beads (Beckman Coulter, Brea, CA) and subjected to real-time PCR analysis using RT2 SYBR Green (Qiagen, Germantown, MD) and primers neighboring TT>A polymorphic region (Table S1).

### Luciferase assay

We cloned 340 bp (non-risk) or 339 bp (risk) of DNA sequence surrounding the TT>A polymorphism into a minimal promoter luciferase plasmid, pGLuc-mini-TK (New England BioLab, Ipswich, MA). Each plasmid was transiently co-transfected using FuGene HD (VWR, Radnor, PA) with a pGL3-promoter control plasmid for calculation of transfection efficiency and normalization (gift from Dr. Carol Webb, OMRF). Luciferase assays were performed in HEK293T and THP1 cells. Twenty fours hours post transfection, cells were treated with 1 ug/ml LPS for 24 hours or 50 ng/ml PMA/500 ng/ml ionomycin for 48 hours. To assay enhancer activity, Gaussia luciferase was analyzed from the cell culture media using BioLux GLuc assay kit (New England BioLab). To measure transfection efficiency, cells were lysed and firefly luciferase activity was measured using the Luciferase Assay System (Promega, Madison, WI).

### Affinity purification of nuclear factors

We screened for other proteins that bind to the EMSA probes by biotinylating the oligonucleotides used for EMSA and a scrambled oligonucleotide that served as a negative control (Table S1). Streptavidin magnetic beads (200 ug; Dynalbeads M-280 Streptavidin; Invitrogen) were subjected to two rounds of blocking with 1% BSA in PBS for 15 min and washing with PBS containing 1M NaCl and TE buffer. Biotinlyated oligonucleotides were linked to half the amount of streptavidin beads by incubating for 30 min at room temperature in TE buffer followed by washing with TE buffer. To pre-clear the nuclear extracts of material that could bind non-specifically to the biotinylated oligonucleotides, we incubated the other half of the BSA-blocked beads with 100 ug of nuclear extract in binding buffer (250 mM NaCl, 50 mM Tris Cl, 50% glycerol, 2.5 mM DTT, 2.5 mM EDTA, pH 7.6) containing 15 ng/ul poly dI:dC (Sigma-Aldrich), 0.5 ug/ml BSA, and 0.1% NP40 for 30 min on ice. We then incubated the pre-cleared nuclear extracts with the oligonucleotide-linked Streptavidin beads for 30 min in 37°C water bath with gentle shaking every 5 min, and subsequently washed the products with binding buffer containing 0.1% NP40 three times. The proteins were eluted in 50 ul of 0.2% SDS sample buffer by boiling for 5 min and were then resolved on a Nu-PAGE 4%–12% Bis-Tris gel followed by silver nitrate staining.

### Mass spectrometry analysis

Mass spectrometry analysis was performed using a ThermoScientific LTQ-XL mass spectrometer coupled to an Eksigent splitless nanoflow HPLC system. Bands of interest were excised from the silver nitrate stained Bis-tris gel and destained with Farmer's reducer (50 mM sodium thiosulfate, 15 mM potassium ferricyanide). The proteins were reduced with dithiothreitol, alkylated with iodoacetamide, and digested with trypsin. Samples were injected onto a 10 cm×75 mm inner diameter capillary column packed with Phenomenex Jupiter C18 reverse phase resin. The peptides were eluted into the mass spectrometer at a flow rate of 175 nL/min. The mass spectrometer was operated in a data-dependent mode acquiring one mass spectrum and four CID spectra per cycle. Data were analyzed by searching all spectra that were acquired against the human RefSeq databases using the program Mascot (Matrix Science Inc. Boston, MA). Minimum identification criteria require two peptides with ion scores greater than 50 that are then verified by manual inspection. Western blots were performed to verify the identities of proteins.

### 3C-qPCR assay

We performed the 3C-qPCR assays as described [40] with minor modifications. All cell lines were cultured and harvested in log phase growth. We incubated 1×10^7^ cells in 10 ml of RPMI-1640 culture medium with 1% buffered formaldehyde at room temperature for 10 min. Crosslinking was stopped by adding 1.425 ml of ice cold 1 M glycine. Cells were lysed in 5 ml lysis buffer (10 mM Tris-HCl, pH 7.5; 10 mM NaCl; 5 mM MgCl_2_; 0.1 mM EGTA; Protease and Phosphatase Inhibitor Cocktail Tablets from Roche Applied Science) for 10 min at 4°C. The nuclei were suspended in 500 µl 1.2× restriction buffer [1× Buffer 4; 1× bovine serum albumin (BSA), New England BioLabs (NEB) Inc., Ipswich, MA] containing 0.3% SDS and incubated at 37°C for 1 h with shaking at 900 rpm. The SDS was then sequestered by adding Triton X-100 to 2% and incubating at 37°C for another hour with shaking. One hundred units of the restriction enzyme NlaIII (NEB) were added for a 24 h digestion. The reaction was stopped by adding SDS to 1.6% and incubating at 65°C for 30 min. The digested chromatin was diluted in 6.125 ml of 1.15× ligation buffer (NEB). Residual SDS was sequestered by adding Triton X-100 to 2% and incubating at 37°C for 1 h with shaking. The reaction was then cooled to 16°C and 2000 U of T4 DNA ligase (NEB) were added. After ligation overnight, the chromatin mixture was incubated with 100 mg/ml proteinase K at 65°C overnight to reverse crosslinks. RNA was removed by RNase A (0.5 mg/ml) treatment for 60 min at 37°C. The 3C sample was purified by phenol-chloroform extraction and then amplified by PCR using specific primers listed in Table S1. An enhancer constant primer was designed according to the negative strand of DNA 20 bp downstream of the TT>A polymorphism. A TaqMan probe was designed based on the positive strand DNA sequence located 10 bp downstream of the first NlaIII enzymatic digestion site and 10 bp upstream of the TT>A polymorphism, hybridizing to the opposite strand as compared to the enhancer constant PCR primer. Multiple primers were designed as close as possible to the NlaIII digestion sites in *TNFAIP3* gene region. The primer/probe configurations guarantees that the probe only signals upon extension of the primer across the ligated junction. TaqMan quantitative real-time PCR was performed with TaqMan Universal PCR Master Mix according to the manufacturer's protocol using the following cycling conditions: 50°C for 2 min; 95°C for 10 min; and 45 cycles of 15 s at 95°C and 60 s at 60°C. PCR products were purified using a QIAGEN quick gel purification kit and the sequence of each chimeric DNA was determined by Sanger sequencing. To normalize primer efficiency, control PCR templates were generated by digestion and random ligation of bacterial artificial chromosomes containing *TNFAIP3* gene and the TT>A enhancer (clone RP11-76M10, Empire Genomics, Inc, New York, USA) [41]. A total of 5 µg of BAC clone was digested with NlaIII and then ligated with T4 ligase. The paired primers/probe designed for 3C-qPCR assay were tested on the random ligation product that contains all possible chimeric DNA ligation products in equal molar concentrations.

### Knock down SATB1 by shRNA in HEK293T cells

The SATB1 shRNA and non-silencing shRNA constructs were purchased from SABiosciences, Valencia, CA. HEK293T cells were transiently transfected with SATB1 shRNA construct or control plasmids using the calcium phosphate method. The extent of shRNA-mediated inhibition of SATB1 and its effect on SATB1 expression were evaluated by western blot analysis with anti-SATB1 antibody. Protein expression of A20 was determined using Western blot with anti-A20 antibody.

### Allele specific 3C PCR sequencing

3C was performed on EBV-transformed B cell lines heterozygous for the TT>A variant as previously described. Chimeric DNA generated by ligation was amplified by PCR and subject to gel purification with a DNA purification kit (QIAGEN Inc., Valencia, CA). Sequencing libraries were constructed using the Truseq DNA LT Sample Prep Kit v2 as per the manufacturer's protocol (Illumina, San Diego, CA). Sequencing of indexed library pools was performed on an Illumina MiSeq instrument with 100 bp, paired-end reads. Reads were mapped to the human reference genome (hg19) using the Burrows Wheeler Aligner (BWA) (Li and Durbin, 2009). The read-count from each allele of the 3C DNA was normalized to the read-count from each allele of genomic DNA (Table S2). A paired *t*-test was used to compare the difference in crosslinking frequencies occurring from two alleles within each individual.

## Supporting Information

Figure S1 The TT>A variants locate in a NF-κB binding site. (a) We used the table browser tool in the UCSC Genome browser to cross reference the TT>A variants (rs148314165, rs200820567) with the ENCODE Integrated Regulation super-track, which contains transcription factor ChIP-seq, H3K4Me1/3 Marks, H3K27Ac Marks, Chromatin State Segmentation, DNaseI Hypersensitivity Clusters, and Vertebrate Conservation data. For transcription factor clusters, the darkness of the segment is proportional to the CHIP-seq signal strength. (b) A zoom in view of the TT>A variants region. The data suggest that the regulatory element is likely to be an enhancer that may interact NF-κB containing nuclear proteins.
(TIFF)

Figure S2 Locations of the three NF-κB binding sites and the SATB1 binding site (a) NF-κB binding sites predicted using UniProbe database are underlined, the TT>A variants are highlighted in red. (b) The locations of the TT>A containing NF-κB site (underline) and the predicted SATB1 site (blue) are shown.
(TIFF)

Figure S3 The TT>A variants result in reduced binding to a nuclear protein complex from THP1 cells that contains NF-κB subunits. Nuclear extracts prepared from THP1 cells were incubated with antibodies against p50, p65, cRel, and SATB1 at room temperature (∼22°C) for 30 min before adding labeled non-risk or risk probes. Antibody against rabbit IgG was used as a negative control. SATB1 antibody demonstrated supershift for both risk and non-risk probes. N.E.: nuclear extract.
(TIFF)

Figure S4 Cold competition demonstrated the EMSA probes specifically bind to the nuclear protein complex contains NF-κB subunits. Nuclear extracts (N.E.) prepared from EBV transformed B cells were incubated with ^32^P labeled non-risk/risk probes, with and without non-risk/risk cold competitors. (a) Labeled probes containing the risk or non-risk sequence were tested for binding affinity with molar excess unlabeled probes also with the risk and non-risk sequence. Unlabeled non-risk probe more effectively competed away the binding of the labeled non-risk probe compared to labeled risk probe as expected. Due to the already low affinity for binding of the labeled risk probe for the nuclear protein complex no definitive competition could be assessed. (b) To further investigate the specificity of the competition, cold probes were then divided up into three small competitors: non-risk 16/risk 15, 5′-16, and 3′-16. Sequences of each competitor are listed in Table S1. As shown in the Figure, only the non-risk 16 probe efficiently competes away the signal as compared to probes: risk 15, 5′-16, and 3′-16.
(TIFF)

Figure S5 Positive and negative controls for ChIP-qPCR assay. ChIP-qPCR assay was performed using EBV transformed B cell lines stimulated with P/I with antibodies against Acetyl-Histone H3 (positive control) and rabbit IgG, followed by qPCR with primers neighboring TT>A polymorphic region. Statistical comparisons were made using one-way ANOVA. Results demonstrated no significant differences in enrichment for either of the controls. N.S.: no significant difference.
(TIFF)

Figure S6 Luciferase activity assay of the regulatory elements carrying the first NF-κB binding site incorporating the TT>A variant in HEK293T cells. 250 bases DNA sequences carrying the variants were cloned into minimal thymidine kinase promoter luciferase construct. The insert DNA includes the only one NF-κB binding site. HEK293T cells were transiently transfected with the above constructs for 24 hours and followed by 48 hours stimulation with P/A, luciferase activity was determined and normalized to internal control vector. Statistical comparisons were performed using a Student's *t*-test of three independent experiments, * indicates p<0.05.
(TIFF)

Figure S7 Western blot and EMSA SS demonstrated that SATB1 binds to the TT>A enhancer region (a) Nuclear extracts from LPS stimulated THP1 cells were incubated with the biotinylated oligonucleotides (non-risk 40/risk 39) bound to streptavidin beads. The bound proteins were eluted and analyzed by SDS-PAGE and silver staining. A scrambled oligonucleotide was used as a negative control. (b) Protein identification by mass spectrometry from 3 independent experiments. (c) Nuclear extracts prepared from no-stimulated and LPS stimulated THP1 cells were incubated with the biotinylated oligonucleotides bound to streptavidin beads. The eluted proteins from streptavidin-oligo beads were analyzed by western blot using anti-SATB1 antibody.
(TIFF)

Figure S8 The TT-A enhancer physically interacted with the TNFAIP3 promoter in multiple lines. 3C-qPCR assays were performed on Daudi, THP-1, Jurkat, and HEK293T cells, relative crosslinking frequencies were normalized to BAC clone control, as detailed in the method section.
(TIFF)

Table S1 List of primers for EMSA, cold competition, ChIP-qPCR, and 3C. Bold text in EMSA probes indicates the NF-κB binding site; underline text indicates the binding sequence of SATB1. Primers producing positive signals in 3C-qPCR assays are marked with an asterisk.
(TIF)

Table S2 Read-count from 3C and genomic DNA input.
(TIFF)
