# Supplementary figures and images for: An Enhancer Element Harboring Variants Associated with Systemic Lupus Erythematosus Engages the TNFAIP3 Promoter to Influence A20 Expression
Source: PLoS Genet. 2013 Sep 5;9(9):e1003750. doi: 10.1371/journal.pgen.1003750 (PMC3764111; doi:10.1371/journal.pgen.1003750)

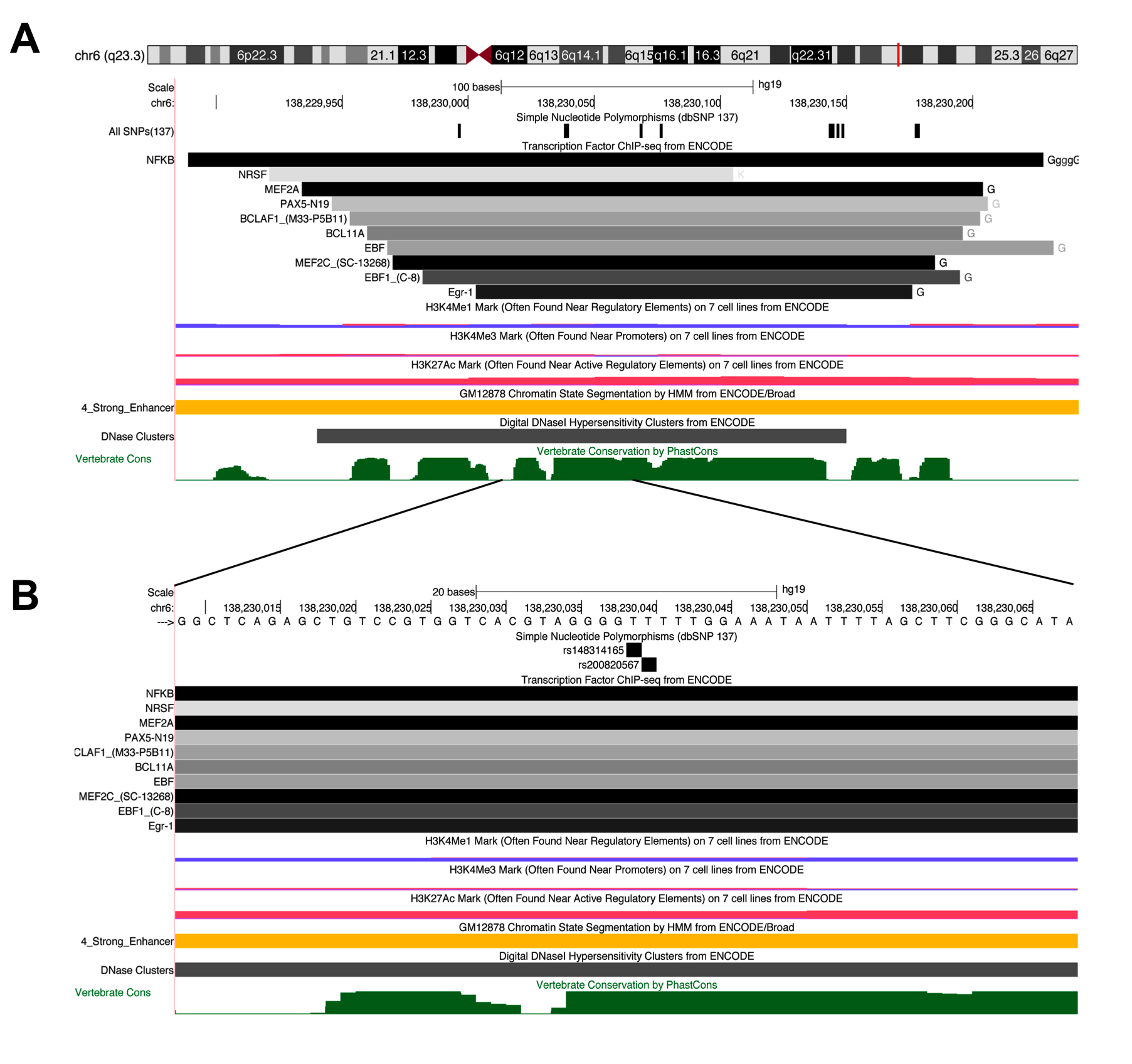

Supplement: Figure S1 — The TT>A variants locate in a NF-κB binding site. (a) We used the table browser tool in the UCSC Genome browser to cross reference the TT>A variants (rs148314165, rs200820567) with the ENCODE Integrated Regulation super-track, which contains transcription factor ChIP-seq, H3K4Me1/3 Marks, H3K27Ac Marks, Chromatin State Segmentation, DNaseI Hypersensitivity Clusters, and Vertebrate Conservation data. For transcription factor clusters, the darkness of the segment is proportional to the CHIP-seq signal strength. (b) A zoom in view of the TT>A variants region. The data suggest that the regulatory element is likely to be an enhancer that may interact NF-κB containing nuclear proteins. (TIFF) [file pgen.1003750.s001.tiff]

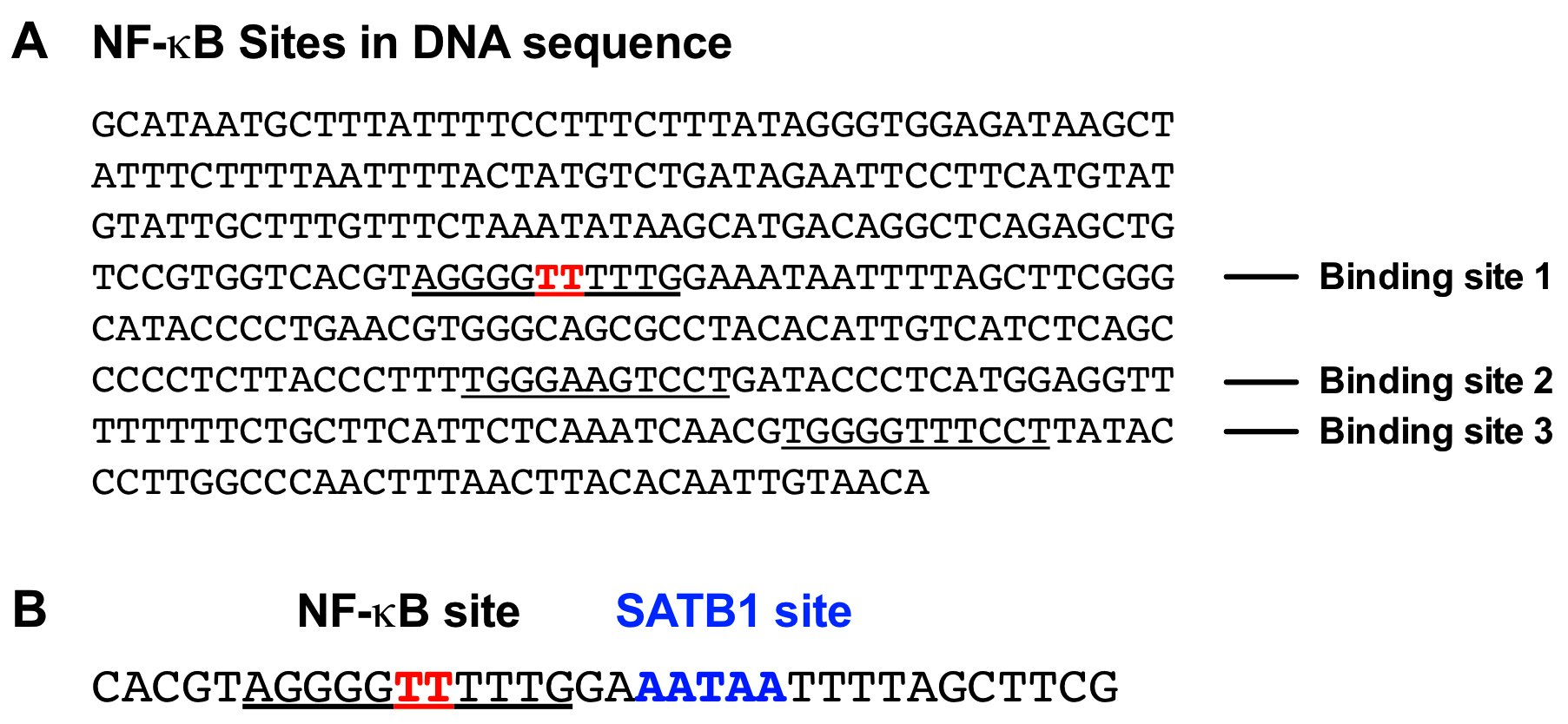

Supplement: Figure S2 — Locations of the three NF-κB binding sites and the SATB1 binding site (a) NF-κB binding sites predicted using UniProbe database are underlined, the TT>A variants are highlighted in red. (b) The locations of the TT>A containing NF-κB site (underline) and the predicted SATB1 site (blue) are shown. (TIFF) [file pgen.1003750.s002.tiff]

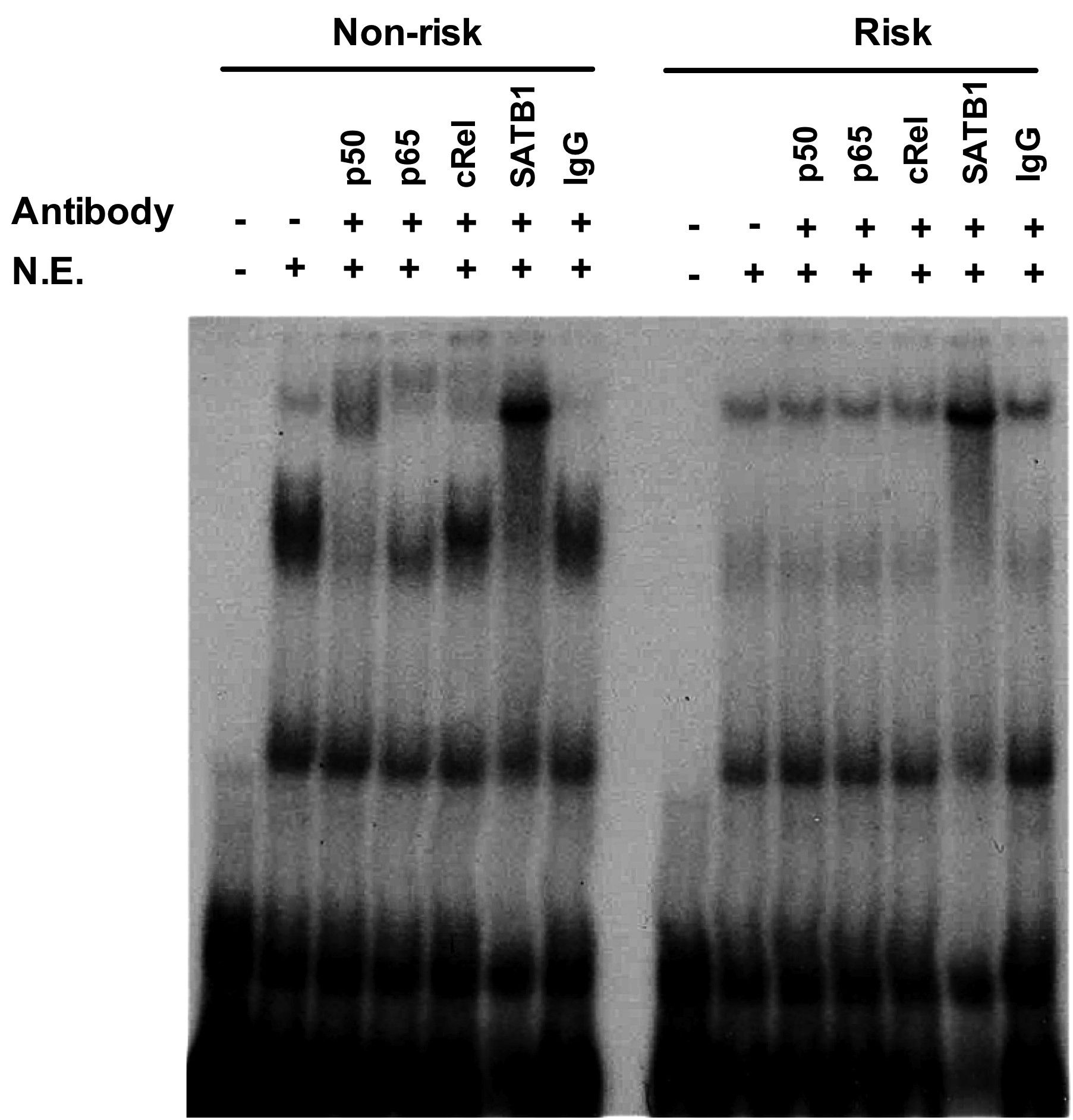

Supplement: Figure S3 — The TT>A variants result in reduced binding to a nuclear protein complex from THP1 cells that contains NF-κB subunits. Nuclear extracts prepared from THP1 cells were incubated with antibodies against p50, p65, cRel, and SATB1 at room temperature (∼22°C) for 30 min before adding labeled non-risk or risk probes. Antibody against rabbit IgG was used as a negative control. SATB1 antibody demonstrated supershift for both risk and non-risk probes. N.E.: nuclear extract. (TIFF) [file pgen.1003750.s003.tiff]

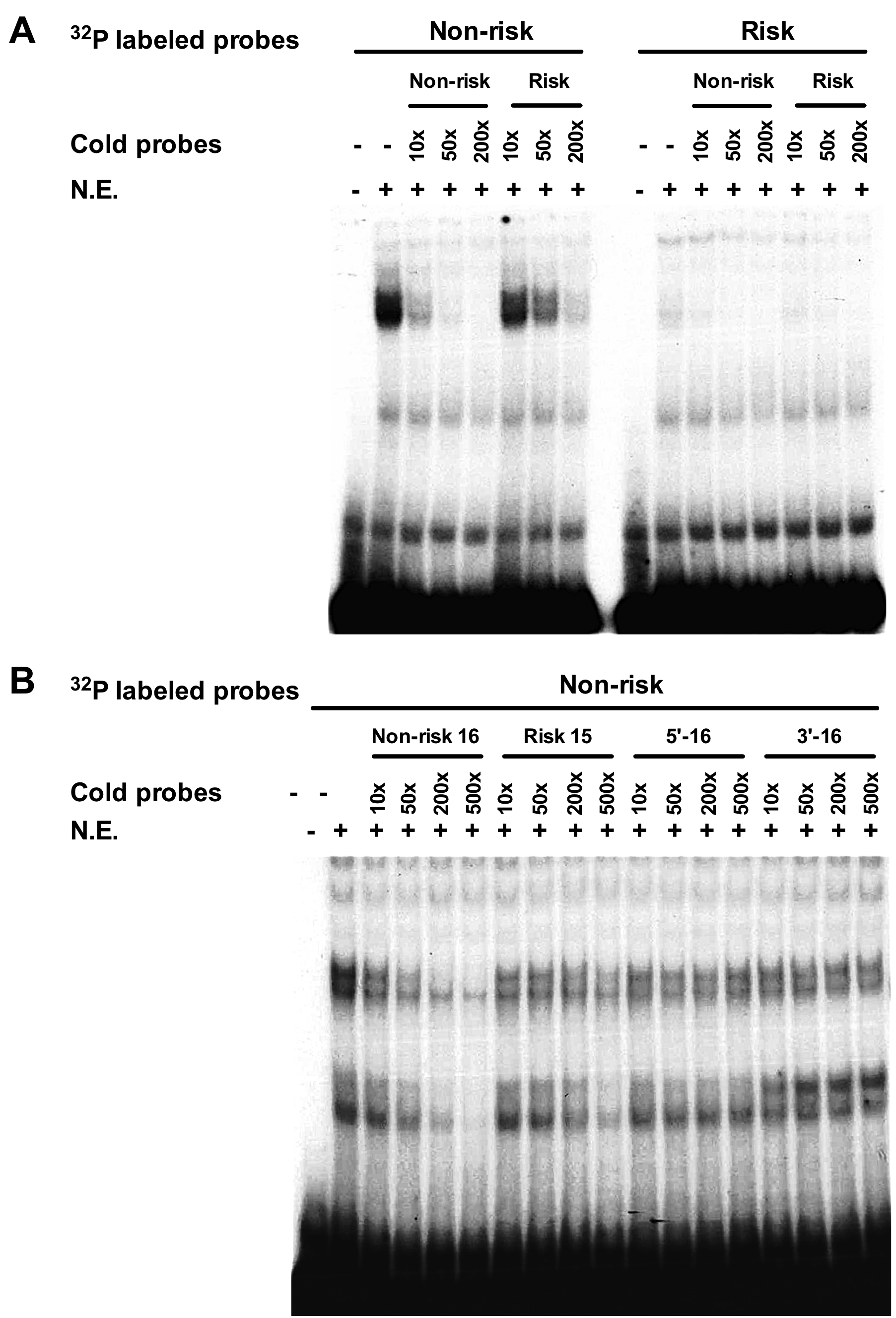

Supplement: Figure S4 — Cold competition demonstrated the EMSA probes specifically bind to the nuclear protein complex contains NF-κB subunits. Nuclear extracts (N.E.) prepared from EBV transformed B cells were incubated with 32P labeled non-risk/risk probes, with and without non-risk/risk cold competitors. (a) Labeled probes containing the risk or non-risk sequence were tested for binding affinity with molar excess unlabeled probes also with the risk and non-risk sequence. Unlabeled non-risk probe more effectively competed away the binding of the labeled non-risk probe compared to labeled risk probe as expected. Due to the already low affinity for binding of the labeled risk probe for the nuclear protein complex no definitive competition could be assessed. (b) To further investigate the specificity of the competition, cold probes were then divided up into three small competitors: non-risk 16/risk 15, 5′-16, and 3′-16. Sequences of each competitor are listed in Table S1. As shown in the Figure, only the non-risk 16 probe efficiently competes away the signal as compared to probes: risk 15, 5′-16, and 3′-16. (TIFF) [file pgen.1003750.s004.tiff]

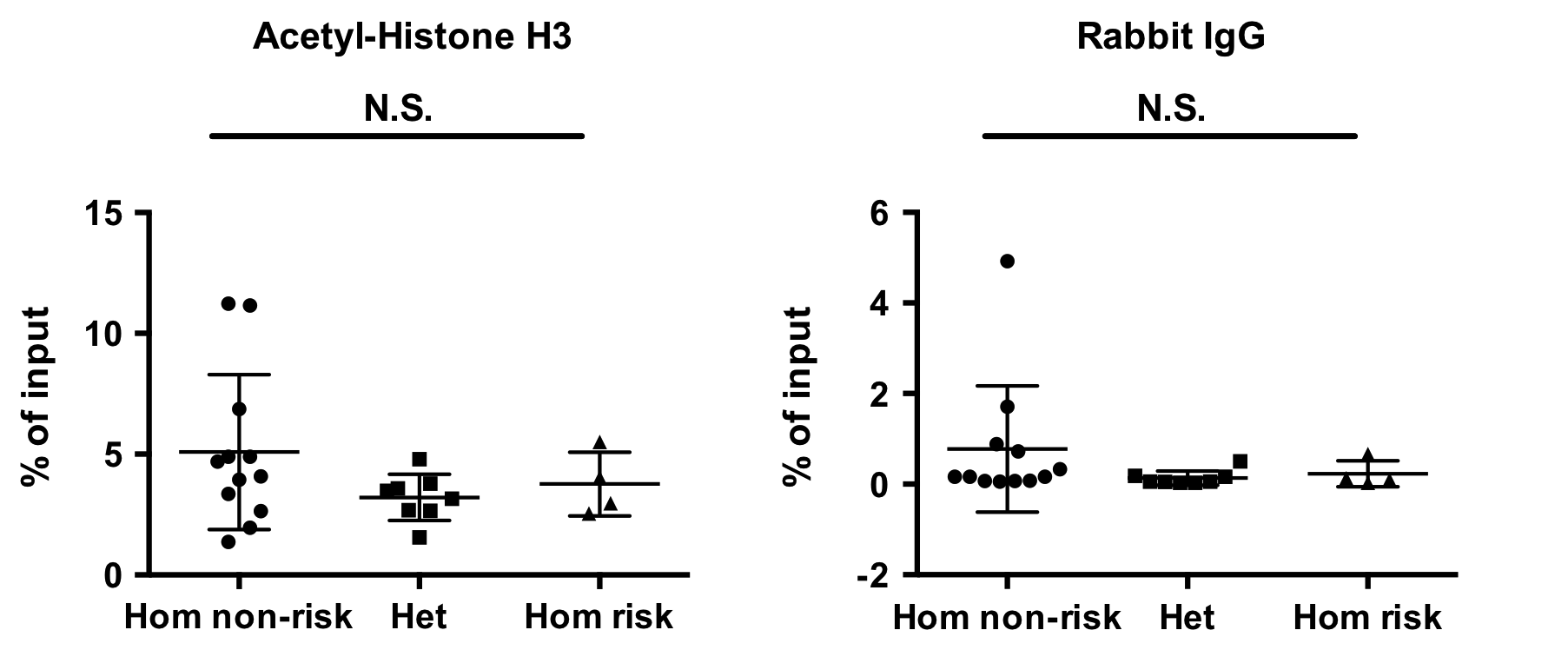

Supplement: Figure S5 — Positive and negative controls for ChIP-qPCR assay. ChIP-qPCR assay was performed using EBV transformed B cell lines stimulated with P/I with antibodies against Acetyl-Histone H3 (positive control) and rabbit IgG, followed by qPCR with primers neighboring TT>A polymorphic region. Statistical comparisons were made using one-way ANOVA. Results demonstrated no significant differences in enrichment for either of the controls. N.S.: no significant difference. (TIFF) [file pgen.1003750.s005.tiff]

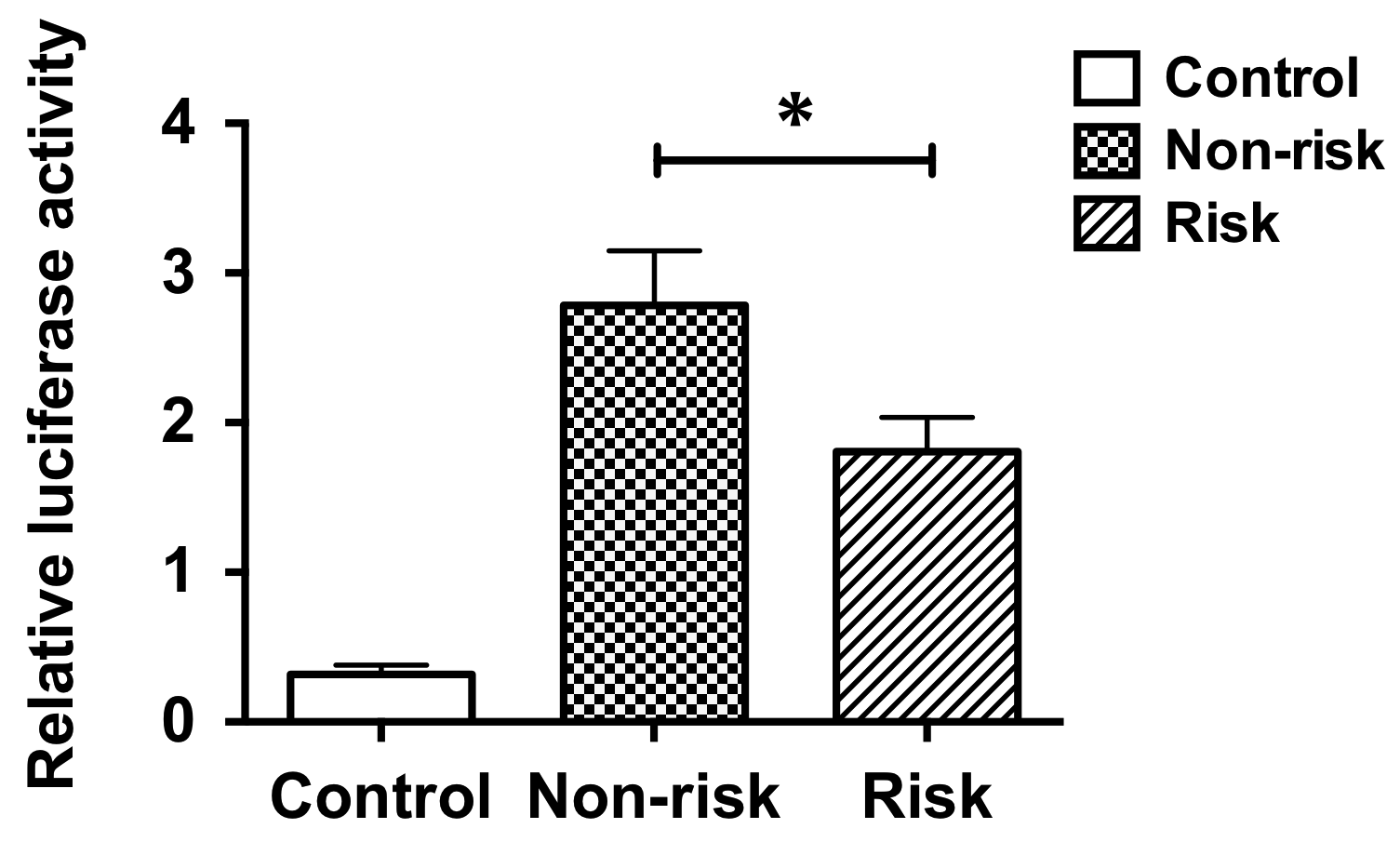

Supplement: Figure S6 — Luciferase activity assay of the regulatory elements carrying the first NF-κB binding site incorporating the TT>A variant in HEK293T cells. 250 bases DNA sequences carrying the variants were cloned into minimal thymidine kinase promoter luciferase construct. The insert DNA includes the only one NF-κB binding site. HEK293T cells were transiently transfected with the above constructs for 24 hours and followed by 48 hours stimulation with P/A, luciferase activity was determined and normalized to internal control vector. Statistical comparisons were performed using a Student's t-test of three independent experiments, * indicates p<0.05. (TIFF) [file pgen.1003750.s006.tiff]

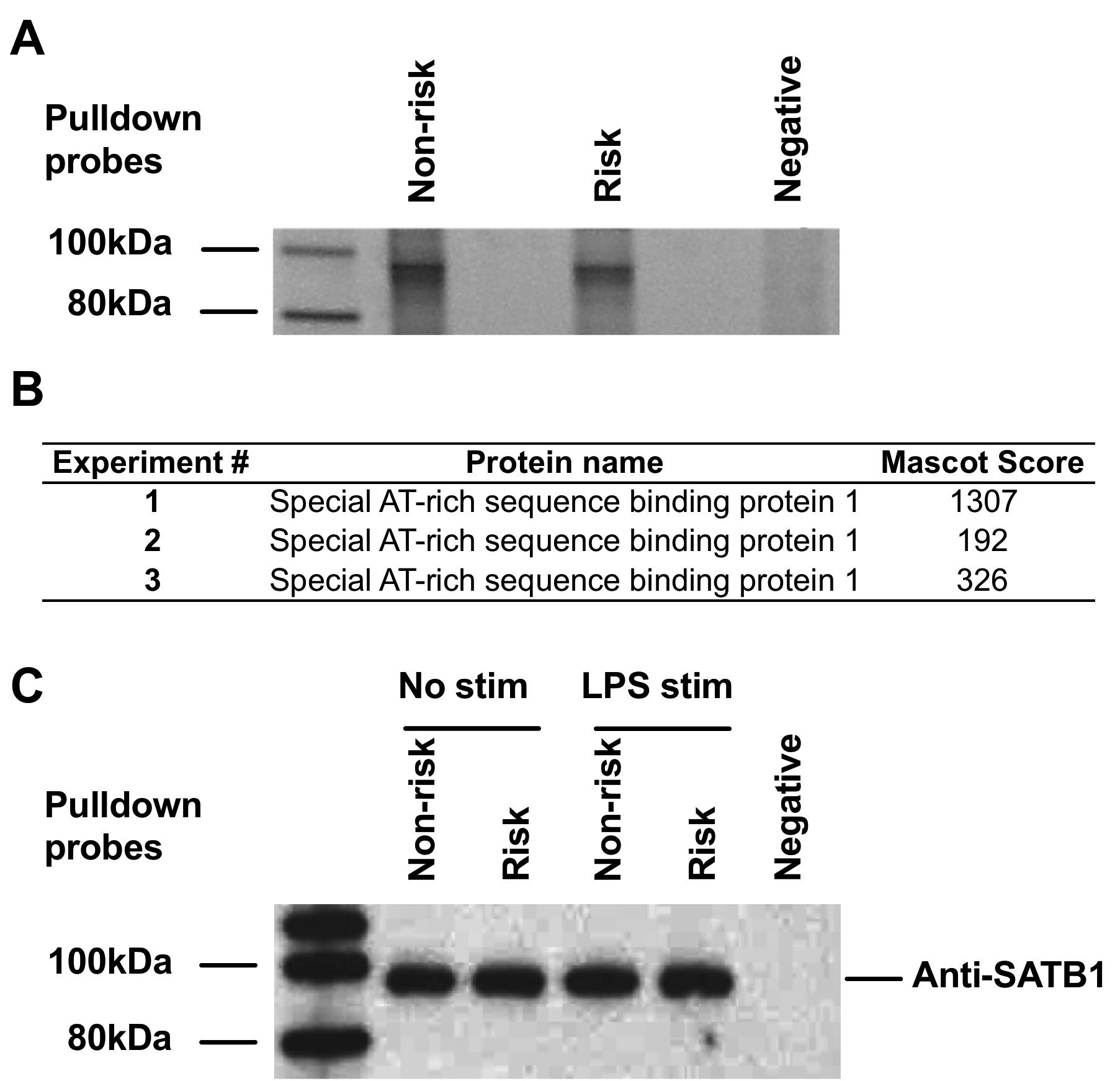

Supplement: Figure S7 — Western blot and EMSA SS demonstrated that SATB1 binds to the TT>A enhancer region (a) Nuclear extracts from LPS stimulated THP1 cells were incubated with the biotinylated oligonucleotides (non-risk 40/risk 39) bound to streptavidin beads. The bound proteins were eluted and analyzed by SDS-PAGE and silver staining. A scrambled oligonucleotide was used as a negative control. (b) Protein identification by mass spectrometry from 3 independent experiments. (c) Nuclear extracts prepared from no-stimulated and LPS stimulated THP1 cells were incubated with the biotinylated oligonucleotides bound to streptavidin beads. The eluted proteins from streptavidin-oligo beads were analyzed by western blot using anti-SATB1 antibody. (TIFF) [file pgen.1003750.s007.tiff]

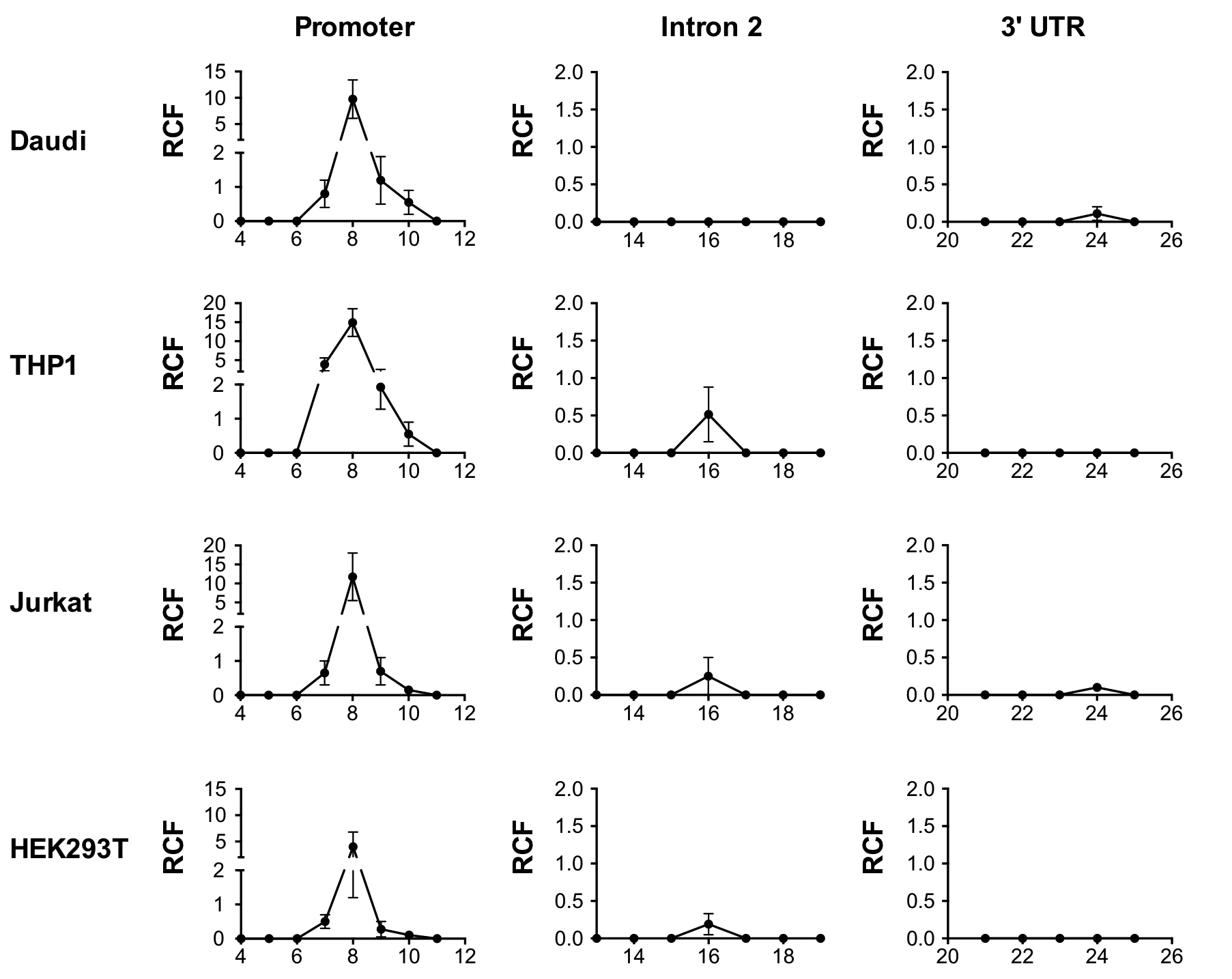

Supplement: Figure S8 — The TT-A enhancer physically interacted with the TNFAIP3 promoter in multiple lines. 3C-qPCR assays were performed on Daudi, THP-1, Jurkat, and HEK293T cells, relative crosslinking frequencies were normalized to BAC clone control, as detailed in the method section. (TIFF) [file pgen.1003750.s008.tiff]

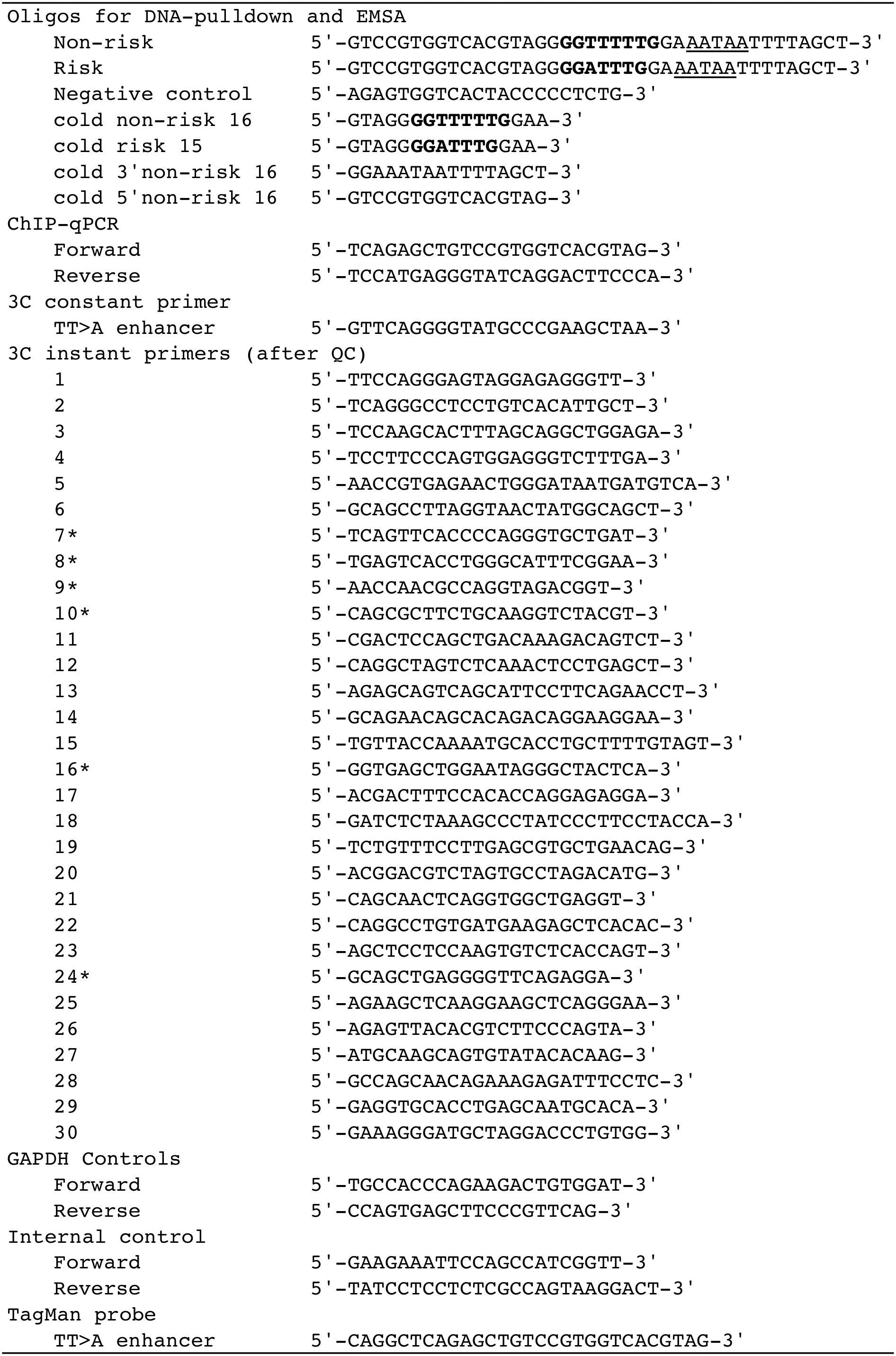

Supplement: Table S1 — List of primers for EMSA, cold competition, ChIP-qPCR, and 3C. Bold text in EMSA probes indicates the NF-κB binding site; underline text indicates the binding sequence of SATB1. Primers producing positive signals in 3C-qPCR assays are marked with an asterisk. (TIF) [file pgen.1003750.s009.tif]

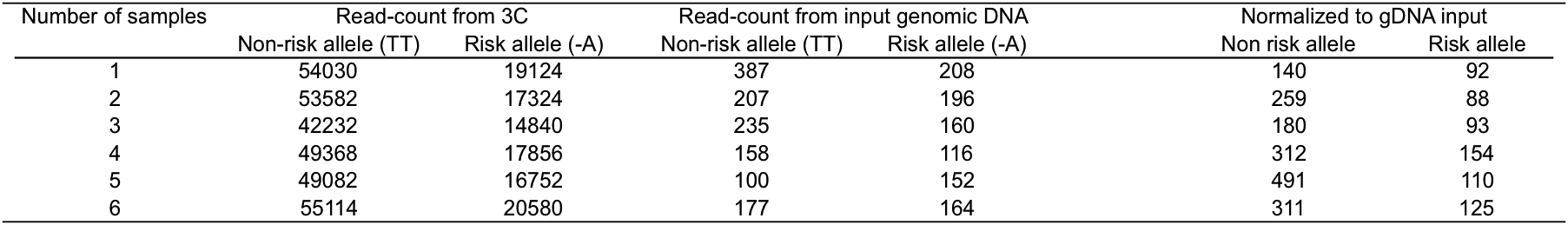

Supplement: Table S2 — Read-count from 3C and genomic DNA input. (TIFF) [file pgen.1003750.s010.tiff]
